# Characterization of Tiled Architecture for C-Band 1-Bit Beam-Steering Transmitarray

**DOI:** 10.3390/s21041259

**Published:** 2021-02-10

**Authors:** Dmitry Kozlov, Irina Munina, Pavel Turalchuk, Vitalii Kirillov, Alexey Shitvov, Dmitry Zelenchuk

**Affiliations:** 1 Nokia Bell-Labs, D15Y6NT Dublin, Ireland; dmitry.1.kozlov@nokia.com; 2 Department of Microelectronics and Radio Engineering, St. Petersburg Electrotechnical University LETI, 197376 St. Petersburg, Russia; ivmunina@etu.ru (I.M.); paturalchuk@etu.ru (P.T.); vvkirillov@etu.ru (V.K.); 3 School of Physics and Astronomy, Cardiff University, The Parade, Cardiff CF24 3AA, UK; ShitvovA@cardiff.ac.uk; 4 ECIT Institute, Queen’s University Belfast, Queen’s Road, Belfast BT3 9DT, UK

**Keywords:** antenna array, antenna measurements, beam pattern, beam steering, equivalent circuit modelling, transmitarray

## Abstract

A new implementation of a beam-steering transmitarray is proposed based on the tiled array architecture. Each pixel of the transmitarray is manufactured as a standalone unit which can be hard-wired for specific transmission characteristics. A set of complementary units, providing reciprocal phase-shifts, can be assembled in a prescribed spatial phase-modulation pattern to perform beam steering and beam forming in a broad spatial range. A compact circuit model of the tiled unit cell is proposed and characterized with full-wave electromagnetic simulations. Waveguide measurements of a prototype unit cell have been carried out. A design example of a tiled 10 × 10-element 1-bit beam-steering transmitarray is presented and its performance benchmarked against the conventional single-panel, i.e., unibody, counterpart. Prototypes of the tiled and single-panel C-band transmitarrays have been fabricated and tested, demonstrating their close performance, good agreement with simulations and a weak effect of fabrication tolerances. The proposed transmitarray antenna configuration has great potential for fifth-generation (5G) communication systems.

## 1. Introduction

Emerging architectures of the fifth-generation (5G) new radio communication systems employ complementary use of both sub-6 GHz and beyond 24 GHz spectrum regions, whereby, in outdoor scenarios, the low-frequency bands are envisioned to provide wide uniform coverage, whereas the millimetre-wave radio would allow directed ultra-high throughput within the wide sub-6 GHz coverage area. Moreover, although millimeter-wave propagation channels exhibit many peculiar features, which may even call for the use of quasi-optical analysis and design techniques, some advanced communication principles and system architectures, primarily aimed at millimetre-wave frequencies, can be implemented and verified with the aid of low-frequency proof-of-concept prototypes.

Multiple-antenna millimeter-wave radio systems, commonly referred to as multiple-input-multiple-output (MIMO) architecture with a large number of antenna elements at the radio access nodes and user terminals enable spatial multiplexing and diversity by means of intelligent beamforming. The latter feature seems to be an indispensable attribute of the 5G communication and radar systems, alongside the exploitation of unconventional degrees of freedom in radio propagation.

Although fully digital beamforming in massive MIMO systems can, in theory, achieve optimal performance, the current state of the digital hardware makes this approach unfeasible for millimeter-wave radio, due to prohibitively high cost and as yet insufficient resolution of the analog/digital-to-digital/analog converters, [1]. On the other hand, fully analog beamforming does not provide essential flexibility in design. In the course of previous studies, it appeared that millimeter-wave channels typically have much less degrees of freedom than can be achieved with fully digital beamforming, thus making the latter redundant. Therefore, many hybrid architectures have emerged recently, aimed to efficiently exploit the sparsity of millimeter-wave channels by combining the key features of both beamforming approaches to achieve optimal performance in applications at reduced complexity and cost.

In particular, the use of refractive dielectric lenses and focusing arrays proved to be technologically advantageous and economically efficient. The use of intelligent reflecting and transmitting surfaces, [1,2], including multi-beam transmitarrays, flat and hybrid lenses, impedance-modulated holographic surfaces, programmable metasurfaces with arbitrary control of the propagated wavefronts, all of which can be realized in conventional planar multi-layer technology using either non-linear materials or surface-mount RF components, opened new avenues in the design of millimeter-wave communication and sensing systems.

Recently, the feasibility of low-bit beam-steering and phase-only beamforming has been demonstrated as a means of further cost-reduction, [3,4]. Beam-switching at the focal-plane array has also been found to be a useful feature for millimeter-wave compact small-cell architectures, [5,6]. A number of different electronically controlled transmitarray architectures have reported recently for applications from C-band to V-band, with various performance functional from merely beam collimation to wide-angle beam-steering, beam-forming and complete wavefront and polarization control. A 28-GHz circularly-polarized reconfigurable transmitarray comprising 400 binary phase unit-cells of receiver-transmitter type with an integrated phase-switch network was experimentally demonstrated in [7] as an attractive solution for many applications operating in Ka-band, such as satellite communications, point-to-point links and heterogeneous wireless networks. The use of a co-designed slot-array focal source antenna enabled a significant reduction of the antenna profile. An X-band electronically reconfigurable transmitarray with enhanced transmission bandwidth and efficiency achieved by using new contactless probe-feeding of the antenna patch was demonstrated in [8], aiming at advanced communication applications. A successful attempt to extend the application of low-cost transmitarrays to V-band was experimentally demonstrated in [9], although no electronic control was available at the time for two-dimensional beam-steering. Most of the above concepts have been demonstrated using integrated transmitarrays fabricated in planar printed-circuit technology. However, fabricating large single-panel transmitarrays raises the cost of proof-of-concept prototyping and makes the technology unaffordable for teaching laboratories.

**Figure 1:**
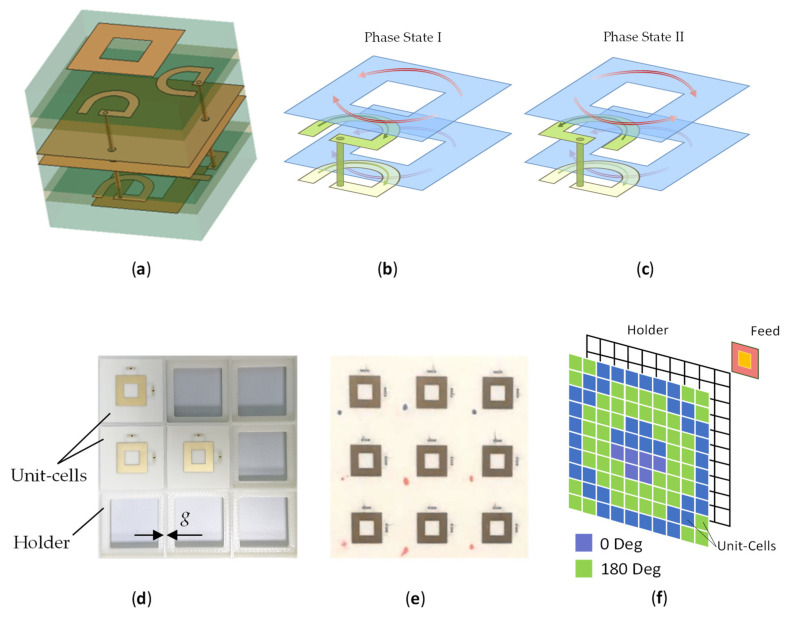
One-bit dual-polarized tiled transmitarray architecture: (**a**) design of the array tile (vertically exploded view), comprising two identical proximity-coupled square-ring radiators on the opposite sides of the tile connected via two U-shaped feed loops; (**b**,**c**) schematic view of the surface currents on the proximity coupled feed loops and square-ring patches in two phase states, respectively (the ground plane is not shown); (**d**) a section of the tiled transmitarray partially assembled; (**e**) a section of the integral single-panel transmitarray; (**f**) proposed device architecture, [10], including individual unit cells (different colors indicate one of the two phase states) to be mounted in the plastic grid frame and spatially fed by a focal-source patch antenna.

Our research is aimed at adopting the transmitarray architecture for MIMO communications in C-band. In our previous publications [10,11], we reported on a low-frequency prototype of novel 1-bit dual-polarized tiled transmitarray, whereby the required phase distribution across the array aperture was built from standalone unit cells manufactured individually and assembled in the required pattern using a rectangular latticed plastic frame, Figure 1. Some preliminary simulation and measurement results were presented, and it appeared that the tiled architecture can be a viable solution for fast prototyping and teaching experiments, without significant performance deterioration, as compared with a similar single-panel transmitarray. Moreover, the possibility of replacing and adding individual elements in the tiled array makes it both repairable and adjustable for a specific focal distance and feed type. This paper revisits previous simulations and provides new results of modelling and experimental characterization of the tiled transmitarray.

## 2. Transmitarray Model

The model of a transmitarray, first presented by the authors in [10], is given below for consistency. In a spatially phase-modulated transmitarray, the normalized wave amplitude received by a unit cell from the focal source reads:

(1) $$a_{mn}=\frac{\lambda e^{𝚥kR_{mn}}}{4\pi R_{mn}}F_{mn}^{fs}\cdot F_{mn}^{ucr}$$

where *m* = 1, 2, …, *M* and *n* = 1, 2,…, *N* are the row and column indexes of the array which define the position of the unit cell with respect to the reference one, k and λ are free-space wavenumber and wavelength, $F_{mn}^{fs}$ is the complex vector field pattern of the focal-plane source transmitting in the direction of the unit cell defined by the corresponding polar and azimuthal angles of the local coordinate system (CS) with the origin at the focal point, $F_{mn}^{ucr}$ is that of the unit cell on receive in the direction of the focal plane source defined by the respective angles of the local CS with the origin at the center of the unit cell (note that the antenna pattern on receive is conjugate of that on transmit due to the reciprocity), the dot symbol denotes the Hermitian inner product of the two complex vector patterns, and $R_{mn}$ is the distance between the focal-source and unit-cell CSs. The unit cells are assumed to be matched to the incoming wave at all angles of incidence determined by the angular aperture of the transmitarray. The effects of the element coupling and finite aperture of the transmitarray can, in principle, be accounted for in the unit-cell antenna patterns by infinite array analysis, [12], or embedded element technique, [13].

The focal-source and unit-cell antenna patterns, in the case of linear polarization, reduce to scalar-valued functions. After sampling and retardation of the incident spherical wavefront, the complex amplitude antenna pattern of the transmitarray, F(θ,ϕ), can be calculated by the pattern multiplication principle, as follows:

(2) $$F\left(\theta ,\phi \right)=\overset{M}{\underset{m=1\;}{\sum }}\overset{N}{\underset{n=1}{\sum }}b_{mn}F_{mn}^{uc}\left(\theta ,\phi \right)e^{𝚥\psi_{mn}^{uc}\left(\theta ,\phi \right)}e^{𝚥kd\left(msin\theta cos\phi +nsin\theta sin\phi \right)}$$

where $F_{mn}^{uc}\left(\theta ,\phi \right)$ and $\psi_{mn}^{uc}\left(\theta ,\phi \right)$ are, respectively, the unit-cell amplitude and phase patterns on transmit, θ and ϕ are azimuthal and polar angles in the spherical CS with the origin at the center of the transmitarray aperture and the polar direction aligned with the transmitarray optical axis, $b_{mn}=T_{mn}a_{mn}$-complex amplitudes of the waves radiated by each unit cell, and $T_{mn}$-the corresponding complex transmission coefficients. Equation (2) enables accounting for the effect of the finite array on the standalone pattern of the element, [14]. Also, the unit-cell radiation pattern is assumed to be independent of the transmission coefficient, i.e., of the specific phase shift for the phase-modulated transmitarray.

The above model can be adopted for the design of the proposed tiled transmitarray by suitably adjusting the unit-cell transmission coefficients for given focal-source and unit-cell antenna patterns. In transmitarray antennas, beam steering is achieved by spatially modulating the phase distribution of the emitted wavefront across the array aperture, as follows:

(3) $$\arg\left(b_{mn}\right)=-k\;r_{s}\cdot r_{mn},$$

where $r_{s}\left(\theta_{s},\phi_{s}\right)$ is the unit vector in the beam-steering direction $\left(\theta_{s},\phi_{s}\right)$, while the array vector $r_{mn}=\left(x_{mn},y_{mn},0\right)$ comprises the coordinates of the unit cell. For symmetrical unit cells, the required continuous local phase shift follows from (1) and (2) as:

(4) $$\arg\left(T_{mn}\right)=\arg\left(b_{mn}\right)-\psi_{mn}^{fs}+kR_{mn}-\psi_{mn}^{uc},$$

where $\psi_{mn}^{fs}$ is the focal-source phase pattern in the direction of the unit cell (NB: typically, the phase pattern, with respect to the phase center of the antenna, is nearly flat within the angular range of the main lobe). In the proposed 1-bit transmitarray, the phase distribution (4) is discretized according to the following recipe (shown for the wrapped phase):

(5) $$\arg\left(T_{mn}^{d}\right)=\{\begin{array}{l} 0{}^{\circ}\;\forall \left|\arg\left(T_{mn}\right)\right|\leq 90{}^{\circ}\\ 180{}^{\circ}\;otherwise \end{array},$$

It is important to note that the effect of the 1-bit phase quantization on radiation characteristics was analyzed in [11,15]. It was shown that a 1-bit resolution results in the antenna gain reduction of up to 4 dB, higher sidelobe level and noticeable beam squint.

## 3. Unit Cell Design and Characterization

The detailed description of the unit cell design and preliminary results of the measurements inside the rectangular waveguide were reported in [10]. This unit cell structure has been employed in the current study. It is noteworthy that the proposed unit cell structure was conceived as a blank of a reconfigurable pixel of single-panel transmitarrays, using surface-mount solid-state switches to add functionality. However, in the context of the tiled transmitarray, power routing is much more challenging and thus is not addressed in this work.

The unit cell design, first reported in [10], was implemented in a stacked 6-layer structure, Figure 1a. The receiving and transmitting antennas were represented by square-ring microstrip elements with electromagnetic (proximity coupled) feeds in the form of open-ended half-wavelength semi-annular (U-shaped) microstrip loops in the layer beneath the square-ring antenna. The proximity coupling allowed a wider bandwidth when the feed loop and ring were properly aligned, [16]. The track widths of the square ring and feed loops were numerically optimized for the maximum return-loss bandwidth and low insertion loss, using CST Microwave Studio simulations with Floquet periodic boundary conditions (FPBCs) and assuming infinite ground plane. The pair of loop resonators were connected to each other by a buried via hole.

The receiving and transmitting sides of the tiled unit cell were separated by two ground plane electrodes bonded together using a 0.2 mm layer of Rogers RO4350B and protruded by the buried vias. The redundancy of the two ground planes was imposed by the manufacturing process. The metallic patterns of the square-ring radiators and feed loops were formed on and between dielectric layers of 0.51 mm thick Rogers RO4003 material (dielectric constant Dk = 3.5, dissipation factor Df = 0.0018). The layers of 0.1 mm bonding film Rogers RO4003C (Dk = 3.38) were used to stack the RO4003 layers. The lateral size of the unit cell of the single-panel transmitarray was 24 mm × 24 mm (~0.46 λ at the design frequency of 5.75 GHz) and its thickness was <0.045 λ. The tiled unit cells were trimmed by 0.5 mm around the edge in order to keep the same array period in both single-panel and tiled transmitarrays.

In the proposed unit-cell design, a 180° phase shift is implemented by switching the feed point of the U-shaped resonator on the receiving side of the transmitarray, Figure 1b,c. The state when the resonators at the receiving and transmitting sides are connected such that the currents flowing in the patches are codirectional is referred to as the phase state I (or 0° state). In the reciprocal phase state II, the resonators are connected at the opposite ends, so that the currents flow in the opposite directions thus imparting a 180° phase shift with respect to the phase state I. Two pairs of feed loops are used on each side of the structure, placed orthogonal to each other so that the unit cell can support two orthogonal linear polarizations for each phase state. The transmission and reflection coefficients measured in the waveguide were similar in both phase states and for both polarizations. The 10 dB return-loss bandwidth spanned 160 MHz from 5.67 to 5.83 GHz. The differential phase error did not exceed ±6° across the operating band.

The transmitarray design approach adopted in our study is based upon the unit cell characterization in terms of insertion loss and differential phase shift (i.e., the phase shift in one phase state with respect to the other)-numerical with full-wave electromagnetic simulations (CST Microwave Studio), as well as experimental inside a rectangular waveguide. The tiled transmitarray has slotted dielectric substrate and ground plane, as well as additional dielectric frame 3D-printed in ABS (acrylonitrile butadiene styrene, Dk = 2.35 as measured), see Figure 1d, necessary to arrange the tiles in desired planar phase pattern. Thus, the effect of the discontinuity, i.e., the width of the gap between the adjacent unit cells, on the tile radiation performance is inherent to the design of the tiled transmitarray and we aimed to minimize its impact within the operating band.

**Figure 2:**
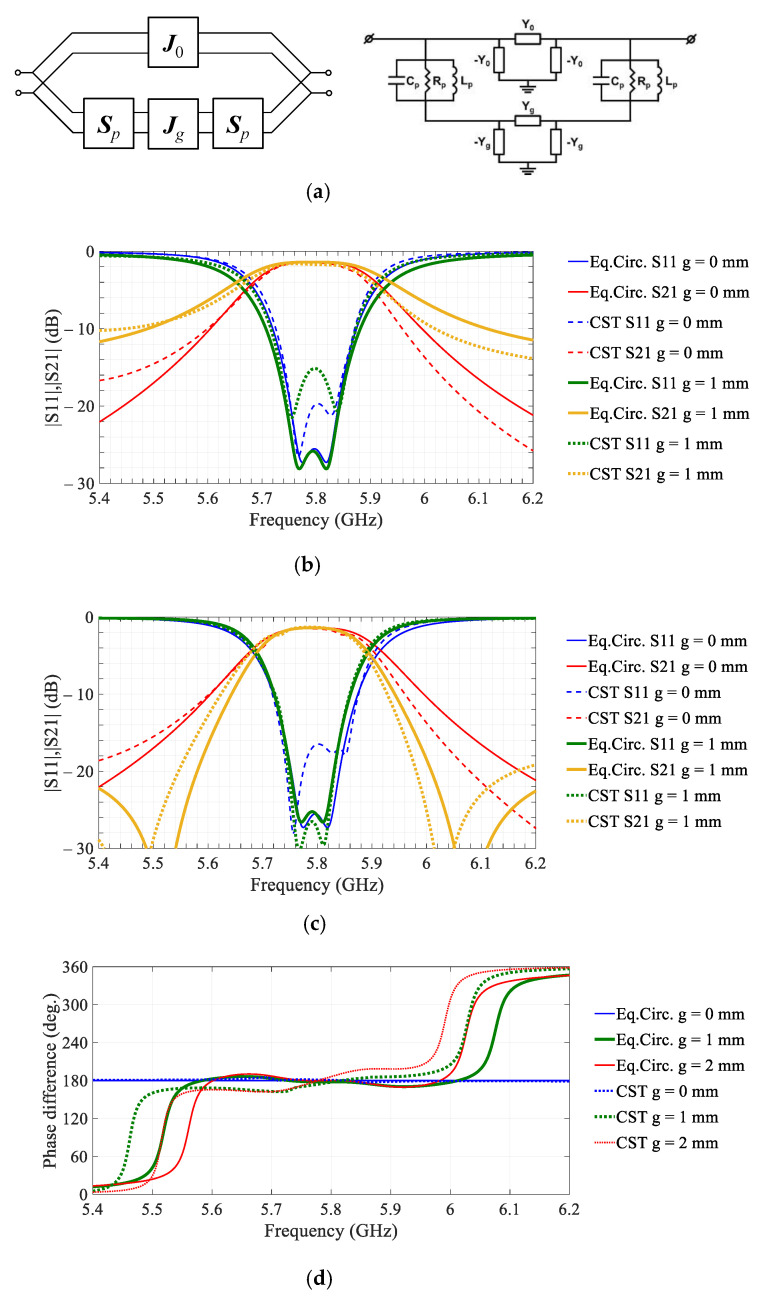
Equivalent-circuit modelling of the unit cells of the single-panel (*g* = 0 mm) and tiled (*g* = 1 or 2 mm) transmitarrays: (**a**) network topology (left) and compact electrical circuit model (right); (**b**) comparison of the circuit model with full-wave electromagnetic simulations (CST Microwave Studio) for the phase state I; (**c**) same as (**b**) but for the phase state II; (**d**) effect of the edge gap width, *g*, on the differential phase shift.

The effect can be elucidated with the aid of the compact circuit model of the tiled unit cell shown in Figure 2a. It is noteworthy that the circuit model is loosely related to the actual geometry of the unit cell and it is derived essentially by emulating the bandpass response of the unit cell in the two phase-states. Nevertheless, the compact model provides useful insights on the interactions of different parts of the unit cell structure.

The circuit model topology constitutes a canonical parallel–parallel connection of the cascaded two-ports, Figure 2a. Being reduced to equivalent elements, the circuit comprises two parallel RLC-circuits ($R_{p}$, $L_{p}$, and $C_{p}$) associated with the receiving and emitting square-ring patches loaded by the respective U-shaped resonators and coupled via the two ideal admittance inverters, $J_{0}$ and $J_{g}$, with characteristic admittances $Y_{0}$ and $Y_{g}$, respectively. The circuit model differs for the two phase states, due to the opposite direction of the current flowing on the receiving patch and this difference is implemented by changing the sign of the inverter admittance $Y_{0}$, with its positive value corresponding to the phase state I and negative to the phase state II. The $J_{0}$-inverter represents the primary coupling of the patches by the via connection, see Figure 1a. The effects of the edge gap are modelled by the additional inverter with a characteristic admittance $Y_{g}$, which can accurately model the out-of-band transmission zeros, see Figure 2c.

Putting $R_{p}$ to zero, it can be shown that the transmission zeros appear at frequencies where the following condition fulfils:

(6) $$Y_{0}=-Y_{g}/\left(1-Y_{g}^{2}Z_{p}^{2}\right),$$

where $Z_{p}$ is the complex impedance of the parallel LC circuit.

The resonance nulls in (6) can appear only when $Y_{0}$ and $Y_{g}$ are in phase, due to the negative sign of the denominator in the vicinity of the resonant frequency of the LC circuit. This condition determines the out-of-band 180° steps of the differential phase shifts, demonstrated in Figure 2d when the differential circuit mode with the opposite direction of the currents on the receiving and emitting patches is superseded by the common mode that is driven by the floating ground plane.

The model parameters are shown in Table 1. The parameters were extracted by best-fitting to the full-wave electromagnetic simulations, as follows. Firstly, the initial ‘patch’ circuit parameters $R_{p}$, $L_{p}$, and $C_{p}$ were fitted using the full-wave simulation of the reflection (S11) for the rectangular patch over an infinite ground plane and simplified circuit model without the inverters. In the second step, characteristic admittance of the $Y_{0}$-inverter is extracted by fitting to the full-wave simulations of transmission (S21) of the single-panel transmitarray unit cell (i.e., *g* = 0 mm). Finally, characteristic admittance of the $J_{g}$-inverter is obtained by fitting the model to the full-wave simulations of S21 of the two square-ring patches coupled only through the slotted ground plane, i.e., in the absence of the $J_{0}$-inverter. It appeared that the absolute value of the characteristic admittance $Y_{g}$ decreases for the wider gap.

**Figure 3:**
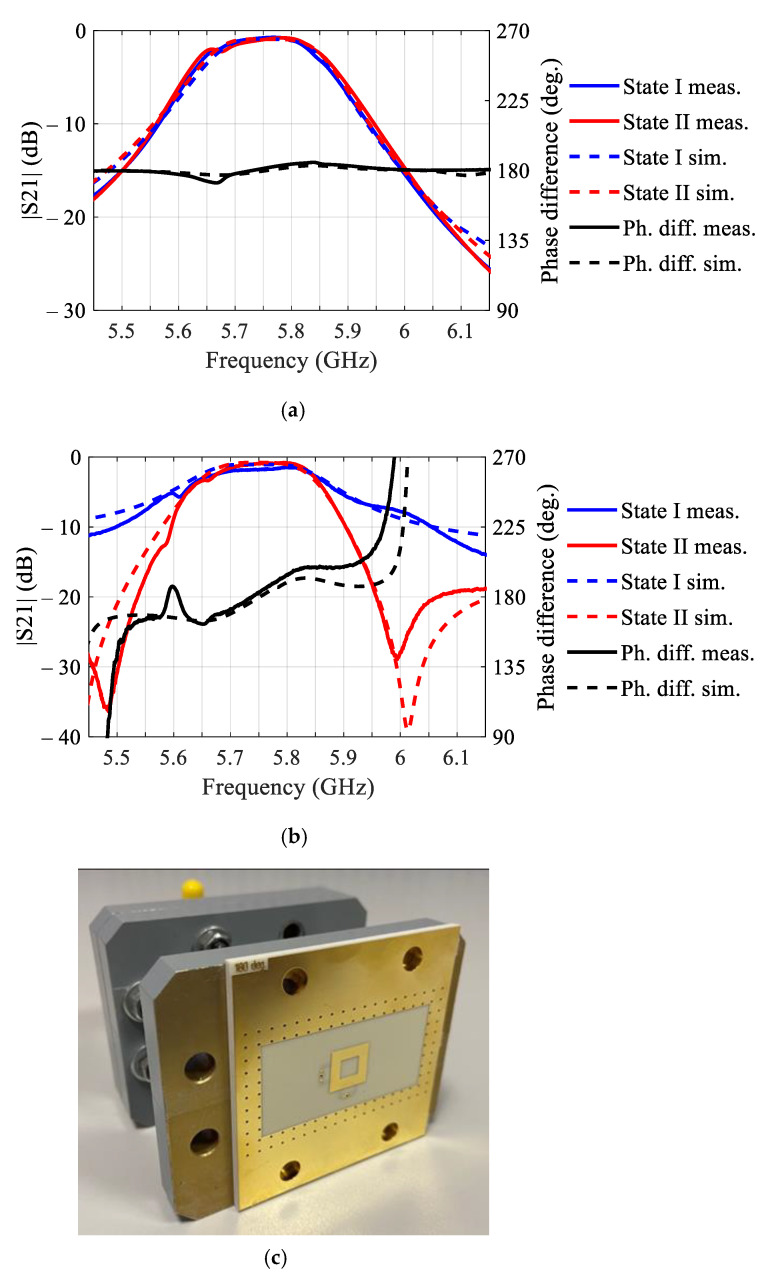
Simulated and measured transmission and differential phase shift of the single-panel without gap (**a**) and tiled with 1 mm gap (**b**) transmitarray unit cells. The results were obtained inside a rectangular waveguide (**c**).

Prototype unit cells emulating the structure of the tiled (1 mm gap width) and single-panel (0 mm gap width) unit cells were fabricated and measured inside the waveguide, see Figure 3a,b respectively. The results in both cases demonstrate noticeable downshift of the central frequency with respect to the design value, c.f., Figure 2b,c, as well as expected degradation of the differential phase shift for the tiled structure, c.f., Figure 2d. The observed shift of the central frequency has been attributed primarily to the specifics of the measurement setup, i.e., different boundary conditions for the unit cell in the waveguide, as compared with the FPBCs in the simulations.

Concluding on the results of characterization of the single-panel and tiled unit cells, it can be noticed that both structures demonstrate similar performance within the operating band. Moreover, two orthogonal polarizations demonstrated close performance, according to the full-wave simulations with FPBCs in [10]. The effects of the gap width can be modelled with a reasonably good accuracy using the compact circuit model in Figure 2a. The beam collimating and steering performances of the transmitarray with specific binary phase distributions are discussed in the next section.

## 4. Beam Steering by the Tiled Transmitarray

Although one may preemptively conclude from the results of the preceding section that the array performance of the tiled architecture should be commensurable with the single-panel transmitarray, there are still important factors yet unaccounted for. Here we shall apply the analytical model (2), alongside the full-wave simulations and antenna measurements, to evaluate the performance of the tiled architecture against the conventional single-panel transmitarray.

Two sets of prototype 10 × 10-element transmitarrays, viz., a set of 120 tiles hard-wired for the two phase states and arranged in specific aperture pattern and a set of three single-panel transmitarrays routed for different beam scan angles (0°, 15°, and 30°), were fabricated in multi-layer printed circuit board technology by two manufacturers using similar materials, but different fabrication processes. The design of the unit cell of the single-panel transmitarrays had to be adjusted to comply with the company-specific fabrication process. That included slightly (10%) decreasing the via diameter and the width of the straight section connecting the annular track of the U-shaped feed to the via, but nevertheless, according to our simulations these changes were not expected to have a prominent effect on the transmitarray performance.

**Figure 4:**
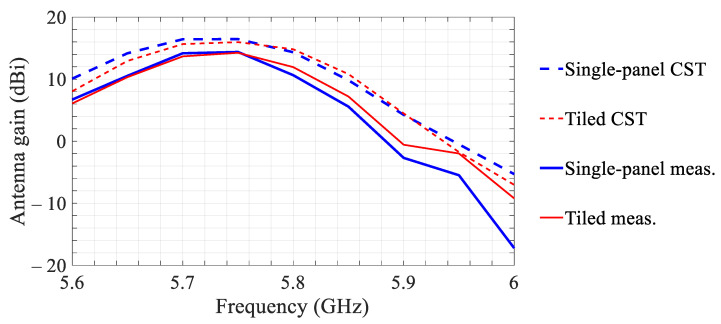
Measured and simulated (with the patch-antenna feed and with the plastic frame in case of the tiled array) boresight gains (H-plane) of the tiled and single-panel transmitarrays versus operating frequency.

The measured and simulated boresight gains (H-plane) versus frequency of the tiled and single-panel transmitarrays illuminated by a patch-antenna feed are shown in Figure 4. The results demonstrate a 3 dB gain bandwidth of 140 MHz from 5.66 to 5.8 GHz for both transmitarrays.

The measured gains in Figure 4 are up to 3 dB lower than the simulated values in the operating band for both transmitarrays, which can be attributed to the simulation accuracy, particularly in estimation of the conductor and dielectric losses, as well as to the fabrication tolerances. Non-uniform amplitude distribution across the transmitarray aperture, due to slightly different transmittance of the unit cells in the two phase-states, might have been another contributing factor. This can be inferred from the measurement results in Figure 3. The measured results also indicate a noticeable (<40 MHz) upshift of the peak-gain frequency of the tiled array with respect to that of the single-panel array. Nevertheless, both arrays demonstrate adequate performance within the operating band.

**Figure 5:**
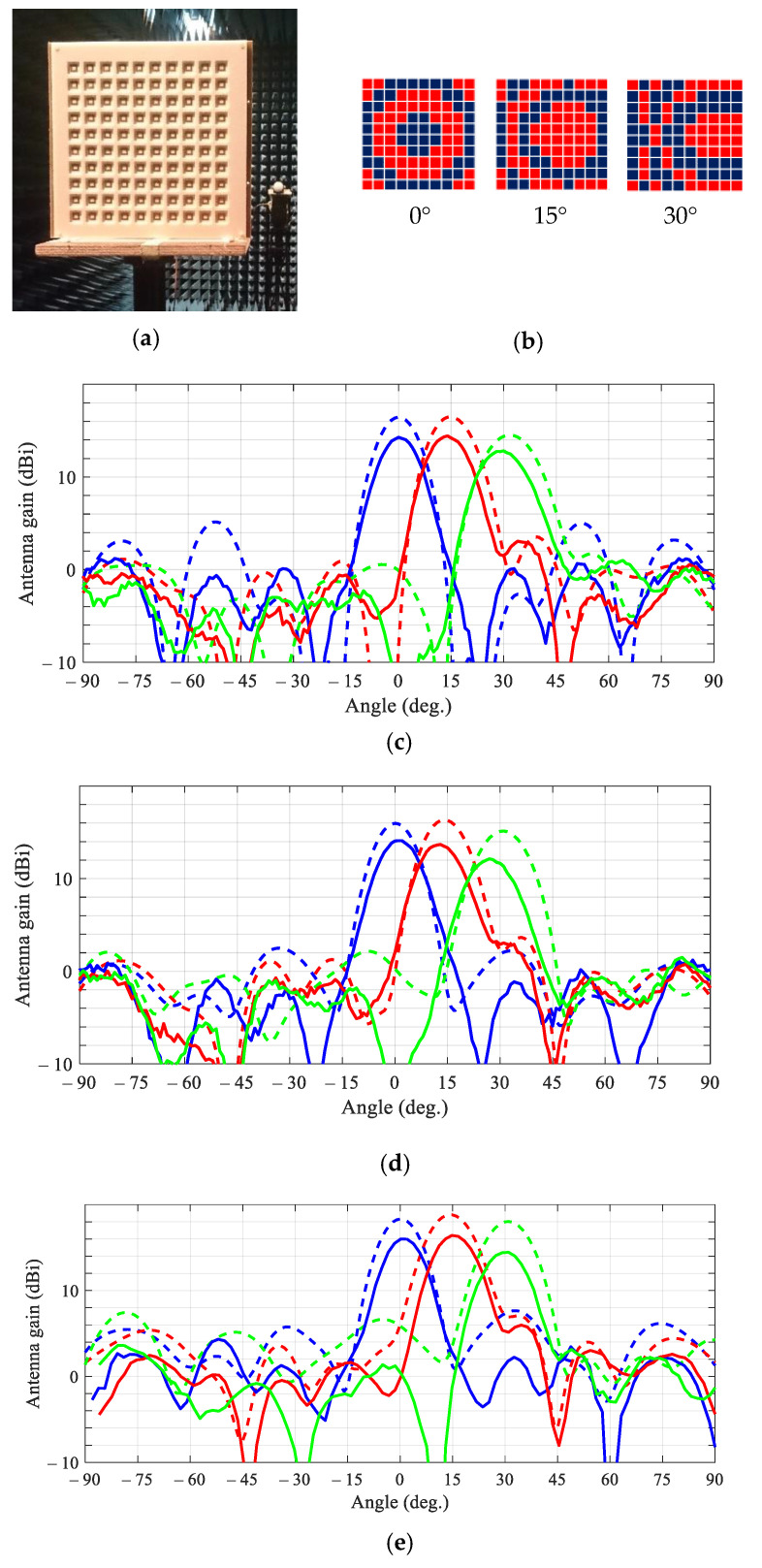
Measured (solid lines) and simulated with the patch antenna feed (dashed lines) beampatterns for different beam-scanning angles (viz., 0°, 15°, and 30°): (**a**) assembled transmitarray with the patch-antenna focal source visible; (**b**) binary phase distribution for different beam-scanning angles; (**c**) H-plane single-panel transmitarray beampatterns; (**d**) H-plane tiled transmitarray beampatterns; (**e**) E-plane tiled transmitarray beampatterns.

The measured and simulated H-plane and E-plane beampatterns of the single-panel and tiled transmitarrays are shown in Figure 5 at the operating frequency of 5.75 GHz. It appears that the tiled array in measurements exhibits a lower gain and a higher beam-pointing error against the simulations, as compared with the single-panel transmitarray. It is noteworthy that the measured 15° beampattern of the single-panel transmitarray and simulated 15° beampattern of the tiled transmitarray feature a higher main lobe as compared with the corresponding central beampatterns. With the aid of the beampattern model (2) we have attributed this feature to the quantization error inherent to the 1-bit phase-shift design, which leads to sub-optimal radiating power combining at boresight of the transmitarray. Deviation of the differential phase shift from 180° causes decreasing peak gain of the steered beams with respect to the central beams in both transmitarrays.

**Figure 6:**
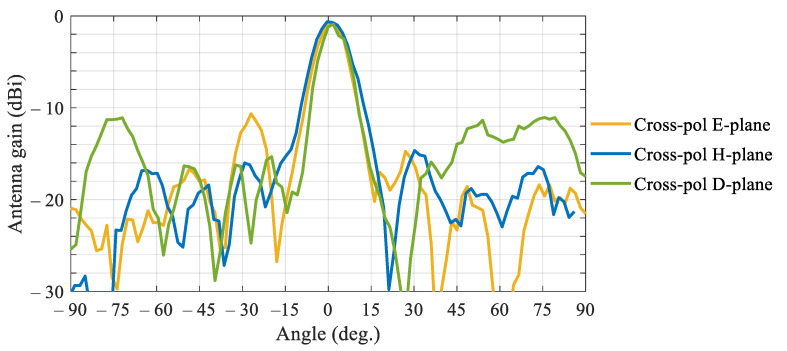
Measured cross-polarization patterns of the tiled transmitarray radiating at boresight plotted in the principal (E and H) and diagonal (D) planes.

Figure 6 shows the measured beam patterns in orthogonal polarization (cross-polarization) in the principal E and H planes and diagonal D-plane. All patterns exhibit a prominent peak at boresight with respective cross-polarization ratio (CPR) ~14.5 dB. The shape of the cross-polarization beampattern is typical for dual-polarization transmitarrays, c.f., [17], and indicates polarization leakage due to coupling between the orthogonal feeds and between the patches on the opposite sides of the unit cell, as indicated by our equivalent-circuit characterisation. The measured figure agrees well with the data reported elsewhere, c.f. [17].

Table 2 shows the performance comparison of the reference single-panel transmitarray discussed in this paper against a selection of published C-band transmitarray implementations, including the theoretical (‘theor.’), measured (‘meas.’) and simulated (‘sim.’) data, [17,18,19,20]. Apart from one passive two-layer frequency-selective surface (FSS) lens, [20], the other transmitarrays adopt the conventional receiver-transmitter architecture with electronic control of the array functional (i.e., beam-steering, beam-forming or polarization conversion). As our design advances, it will integrate electronic control and provide wider bandwidth and better beam pointing accuracy.

## 5. Conclusions

A comprehensive characterization of the tiled transmitarray architecture first proposed in [10] has been carried out in this paper. A new compact circuit model has been devised to analyze the broadband transmission and differential phase shift characteristics of the tiled transmitarray unit cells.

The unit cell characterization at normal incidence has been carried out using the proposed circuit model and full-wave electromagnetic simulations. It appeared that the tiled and single-panel unit cells demonstrate commensurable performance within the operating frequency band, although the tiled unit cell exhibits a higher differential phase error.

The antenna gain and radiation patterns of the fabricated tiled and single-panel transmitarrays have been measured for different beam-scan angles, as well as compared with full-wave electromagnetic simulations. The tiled transmitarray demonstrated slightly lower gain and higher beam-pointing error as compared with the single-panel transmitarray. The measured results are in a good quantitative agreement with simulations.

In conclusion, it has been shown that the tiled transmitarrays can be effectively designed, modelled and fabricated to demonstrate the antenna performance commensurate with conventional single-panel transmitarrays. Considering the cost of manufacture and flexibility in configuring the transmitarray for various applications, the proposed tiled transmitarray architecture proves to be a feasible and economically effective solution for 5G communication systems. The future work will be carried out on advancing the analytical model by taking into account essential effects due to spillover, [21], coupling and array non-uniformity, adopting the tiled architecture for millimeter-wave applications, investigating the heterogeneous and conformal transmitarrays enabled by the tiled architecture, as well as developing hybrid approaches to beam-scanning and beam-forming by combining tiled transmitarrays with focal plane antenna arrays.

## Figures and Tables

**Table 1:** Parameters of the compact circuit model of the transmitarray unit cell extracted by fitting to the full-wave simulations (see Figure 2b,c).

Gap Width (mm)	$R_{p}$ (Ω)	$L_{p}(\mathrm{nH})$	$C_{p}(\mathrm{pF})$	$Y_{0}$ $\left(\Omega^{-1}\right)$	$Y_{g}$ $\left(\Omega^{-1}\right)$
0	300	0.038	19.84	±j0.021	N/A
1	300	0.038	19.86	±j0.021	−j0.23
2	300	0.038	19.89	±j0.021	−j0.18

**Table 2:** Comparison of some existing C-band transmitarrays.

Reference	[17]	[18]	[19]	[20]	This Work (Tiled)
Unit-cell	Two-layer stacked patches (Rx), a patch with O-slot (Tx), reflective phase shifter, vias	Patches, proximity coupled diff. feeds, balanced bridged-T phase shifters	Five stacked layers of square slot FSS and feeding networks	Passive two-layer double split-ring slot unit cells of varying size	Square-ring patch with proximity coupled U-shaped resonators, vias
Array size	8 × 8	6 × 6	5 × 5	7 × 7	10 × 10
Polarization	LP-to-LP/CP	LP	LP	LP	DLP
f0, GHz (Δf/f0)	5.4 (8.5% by AR–3 dB)	5 (10% by G0–2 dB)	5.2 (1.4% by UC S21–3 dB)	6 (15% by G0–3 dB)	5.75 GHz (2.5% by G0–3 dB)
Boresight antenna gain, dBi	17 (meas.)	20.5 (theor.) 15.0 (meas.)	18.6 (sim.) 15.6 (meas.)	16.7 (meas.)	14 (meas.)
HPBW, deg. (meas.)	13.5 (E)	20.4 (E) 18.4 (H)	14 (E) 16 (H)	12	12
SLL, dB (meas.)	−10	−21.1 (E) −14.9 (H)	−9.7	−10	−12
CPR, dB (meas)	20	35	N/A	N/A	14.5
Scan loss (scan angle), dB	0.9 (20 degree) 2.1 (30 degree)	N/A	1.4 (15 degree) 7.8 (25 degree)	N/A	2 (30 degree)
Beam pointing error (H), degree	N/A	N/A	0 (0 degree) 3 (15 degree) 8 (30 degree) 16 (45 degree)	N/A	N/A
Control	Varactors	Varactors	Varactors	None	None

## Data Availability

The data presented in this study are available on request from the corresponding author.
